# Archaeogenetic Evidence of Ancient Nubian Barley Evolution from Six to Two-Row Indicates Local Adaptation

**DOI:** 10.1371/journal.pone.0006301

**Published:** 2009-07-22

**Authors:** Sarah A. Palmer, Jonathan D. Moore, Alan J. Clapham, Pamela Rose, Robin G. Allaby

**Affiliations:** 1 Warwick HRI, The University of Warwick, Wellesbourne, United Kingdom; 2 Worcestershire Historic Environment & Archaeology Service, Woodbury, University of Worcester, Worcester, United Kingdom; 3 The McDonald Institute for Archaeological Research, University of Cambridge, Cambridge, United Kingdom; University College London, United Kingdom

## Abstract

**Background:**

Archaeobotanical samples of barley (*Hordeum vulgare* L.) found at Qasr Ibrim display a two-row phenotype that is unique to the region of archaeological sites upriver of the first cataract of the Nile, characterised by the development of distinctive lateral bracts. The phenotype occurs throughout all strata at Qasr Ibrim, which range in age from 3000 to a few hundred years.

**Methodology and Findings:**

We extracted ancient DNA from barley samples from the entire range of occupancy of the site, and studied the *Vrs1* gene responsible for row number in extant barley. Surprisingly, we found a discord between the genotype and phenotype in all samples; all the barley had a genotype consistent with the six-row condition. These results indicate a six-row ancestry for the Qasr Ibrim barley, followed by a reassertion of the two-row condition. Modelling demonstrates that this sequence of evolutionary events requires a strong selection pressure.

**Conclusions:**

The two-row phenotype at Qasr Ibrim is caused by a different mechanism to that in extant barley. The strength of selection required for this mechanism to prevail indicates that the barley became locally adapted in the region in response to a local selection pressure. The consistency of the genotype/phenotype discord over time supports a scenario of adoption of this barley type by successive cultures, rather than the importation of new barley varieties associated with individual cultures.

## Introduction

Barley (*Hordeum vulgare*) was among the earliest cereal crops to be exploited during the Mesolithic Neolithic transition [1] and the first to become domesticated, about 9500 years before present (BP) [2]. The primary role of barley in the origins of agriculture can be attributed to its resilient qualities such as rapid development to maturation [3], xerophytic adaptations such as long awns [4], as well as tolerance of a wide range of edaphic factors such as pH and salinity [5]. These features made it well suited to the early Holocene climate in which the spring growing season was shorter than it is now, and the summer longer, hotter and drier [3]. Subsequently, barley became the most widespread of the cereal crops being grown in northern extremities of the British Isles and Scandinavia [6], as well as the hot, dry climate of Egypt by the seventh millennium BP [7]. The robust nature of this crop ensures its importance in the future, as crop varieties need to be developed to increase the potential areas amenable to arable agriculture to sustain the expanding human population.

An important architectural feature of barley concerns the spikelet morphology which gives rise to ‘two-row’ and ‘six-row’ forms of the crop. The spikelet is composed of a central floret and two lateral florets. In the two-row forms only the central florets develop grains whereas in six-row both central and lateral florets develop grains. Within these two broad classes a number of morphologies are possible depending on the extent of lateral floret development [8]–[10]. Wild barley (*H. vulgare* ssp. *spontaneum*) occurs in the two-row form, whereas cultivated barley (*H. vulgare* ssp. *vulgare*) has varieties of both two-row and six-row forms. Six-row barley has higher protein content than two-row and is often favoured as a food source as a result [11]. In comparison, the biomass productivity differs little between the two forms due to the ability for compensatory growth, typical of cereals [12]; two-row barleys tend to have fewer, larger grains relative to six-row barleys. However, the two forms have notably different fecundities as a result of the differing number of grains produced; six-row plants typically produce 1.5–2.0 times as many grains as two-row plants [13].

On the African continent six-row barley is mostly grown where it is typically used as a food source, often for animal feed. The archaeological record shows that six-row varieties were available to farmers by 8800 years BP [2] and are present at the earliest African archaeological sites dating to the seventh millennium BP [7], [16] [7], [14].

**Figure 1:**
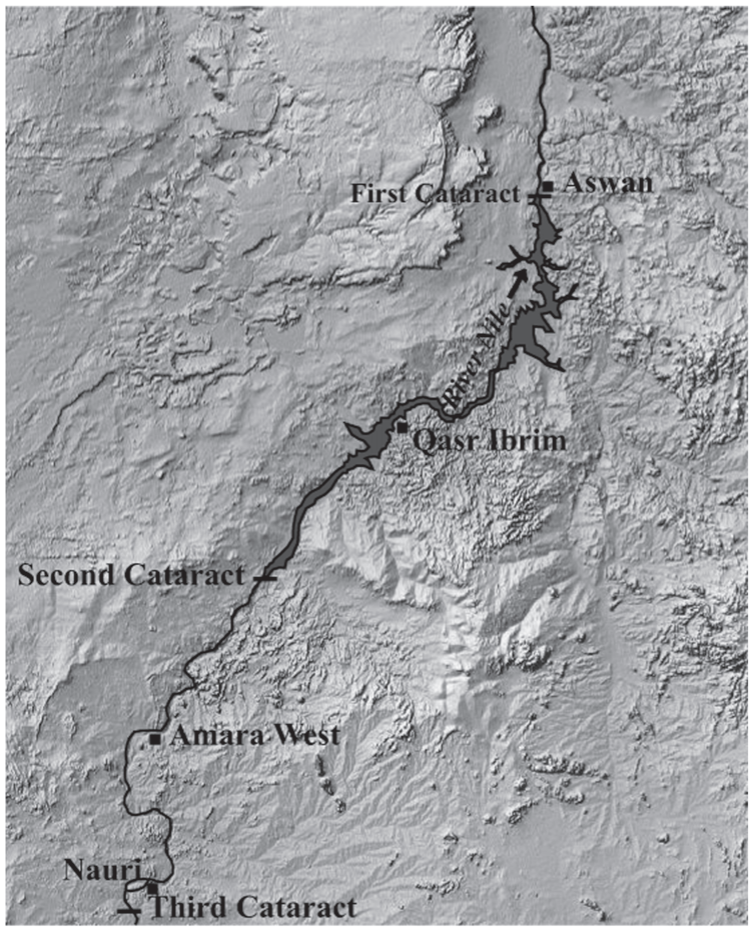
Map of Nubia. Locations of archaeological excavations at Tell el-Amarna, Qasr Ibrim, and Nauri and the cataracts along the River Nile are indicated.

Archaeobotanical remains of barley have been recovered from the archaeological site at Qasr Ibrim (Fig. 1), which was occupied from around 3000 years ago to several hundred years ago [15]. This site is interesting because it was a boundary settlement on the edge of the Nubian and Roman Empires located between the first and second cataracts of the Nile, and was occupied by five successive cultures: Napatan, Roman, Meroitic, Christian and Islamic. Throughout all of the cultural stages the people of Qasr Ibrim grew barley without engineered irrigation but using the natural cycle of hydrology provided by the Nile, so called basin irrigation. The phenotype of archaeobotanical remains of barley from all of the cultural stages at Qasr Ibrim and from Nauri, another archaeological site further upriver of Qasr Ibrim (Fig. 1), is surprising because of its two-row appearance Figure S1, [16]. Contemporaneous archaeobotanical remains found downstream of the first cataract, from sites such as Tell el-Amarna (Fig. 1), are of the six-row type indicating that six-row varieties were available to the people of Qasr Ibrim and indeed were typical of Egypt then as now [14]. Barley was used as animal feed at these sites [15], it is therefore a mystery why the typically preferable 6-row types that were available were not grown, and it is unknown why or how the 2-row phenotype occurred. Did the people of Qasr Ibrim import two-row barley from outside the region? It is a further mystery that this state of affairs was propagated through five successive cultures.

Recently, the genetic mechanism that causes the switch from two to six-row in barley was described [9]. A homeodomain-leucine zipper I-class homeobox gene *Vrs1* produces a transcription factor that inhibits lateral bud growth leading to the two-row condition. The six-row condition is a derived state in which a loss of function mutation occurs in the *Vrs1* gene. Three different loss of function mutations have been identified in the *Vrs1* gene, all of which result in the loss of lateral bud suppression resulting in the six row varieties seen in cultivated barley [9]. Two of these mutations are geographically restricted to the Western Mediterranean and East Asia respectively. The third, defined by the *vrs1.a1* clade in [9], occurs worldwide, is responsible for most of the six-row barley varieties, and is considered the most ancient of the three mutations. The presence of any one of these recessive alleles was found to be sufficient to cause the six-row phenotype by itself [9].

The remarkable biomolecular preservation of archaeobotanical material at Qasr Ibrim makes it highly suited to ancient DNA analysis [17]. In order to investigate the cause of the curious 2-row phenotype present at Qasr Ibrim we amplified the *Vrs1* gene from ancient DNA retrieved from archaeobotanical remains of barley spanning the entire range of occupancy of the site.

## Results and Discussion

**Figure 2:**
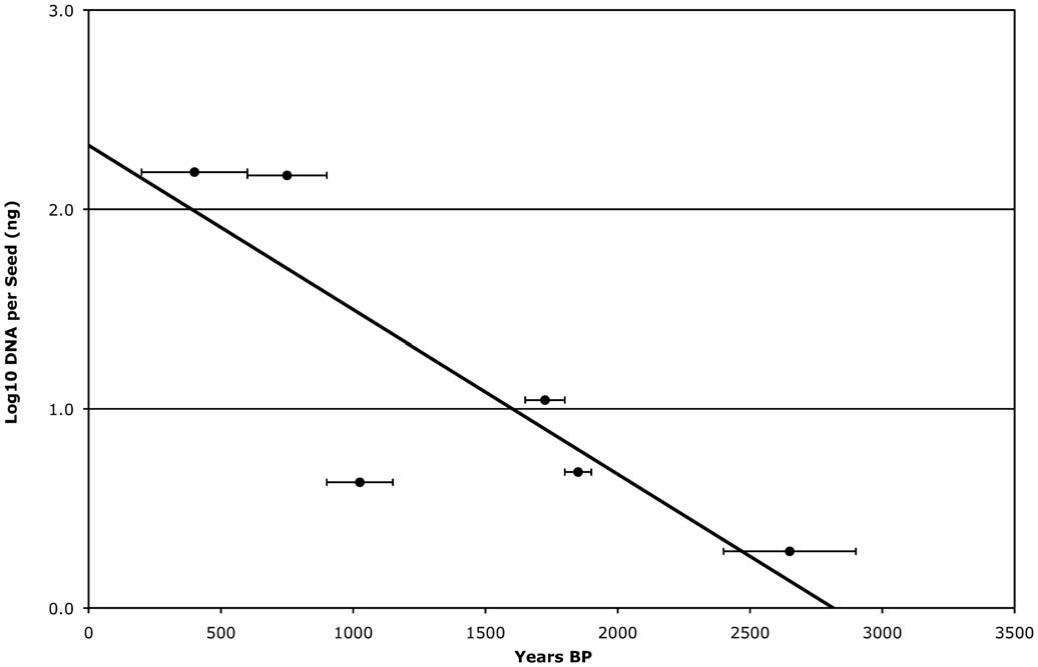
Recoverable archaeological DNA quantity over time. DNA extracted per Barley seed plotted on a logarithmic scale against the years BP of the assemblage from which each sample was recovered. Error bars indicate the range of years BP of each strata from which samples were taken.

Consistent with previous findings from Qasr Ibrim, relatively large amounts of DNA were retrieved from the barley grains (Table S1). The quantities of DNA retrieved appear to decrease in a time dependent manner (Fig. 2). This suggests that the bulk of DNA extracted from these samples is endogenous because one would not expect secondary DNA sources (such as bacteria or human) to correspondingly reduce in amount. The amount of DNA present per seed closely follows an exponential curve with a half-life of approximately 350 years. Under these assumptions we would predict from our empirical data that there would be little chance of retrieving DNA from archaeobotanical remains much older than 3000 years under the preservation conditions at Qasr Ibrim (Figure S2). The principal process of DNA degradation is through hydrolytic depurination. A number of factors influence DNA diagenesis including humidity, pH, temperature and the availability of oxygen [18], [19]. While conditions at Qasr Ibrim are hot, which would serve to speed up oxidative and hydrolytic processes and shorten the preservation time of DNA, they are also very dry, which would greatly reduce the rate of hydrolysis. The preservation of DNA in ancient Egyptian contexts has been the matter of recent debate [20]–[22]. It has been asserted that when Egyptian archaeological sites are subject to occasional flooding or increased humidity caused by flooding, as is the case with some tomb sites, in combination with such high temperatures it is unlikely that DNA would persist for even a few hundred years. Our results are therefore only consistent with a completely dry history at Qasr Ibrim given our knowledge of DNA diagenesis. Qasr Ibrim is a raised settlement, 60 metres above the Nile valley floor and therefore not subject to flooding or increased levels of annual humidity, so it would appear the context is consistent with the preservation. As such, our data may prove a useful empirical baseline in measuring DNA decay in the virtual absence of water at high temperatures.

**Figure 3:**
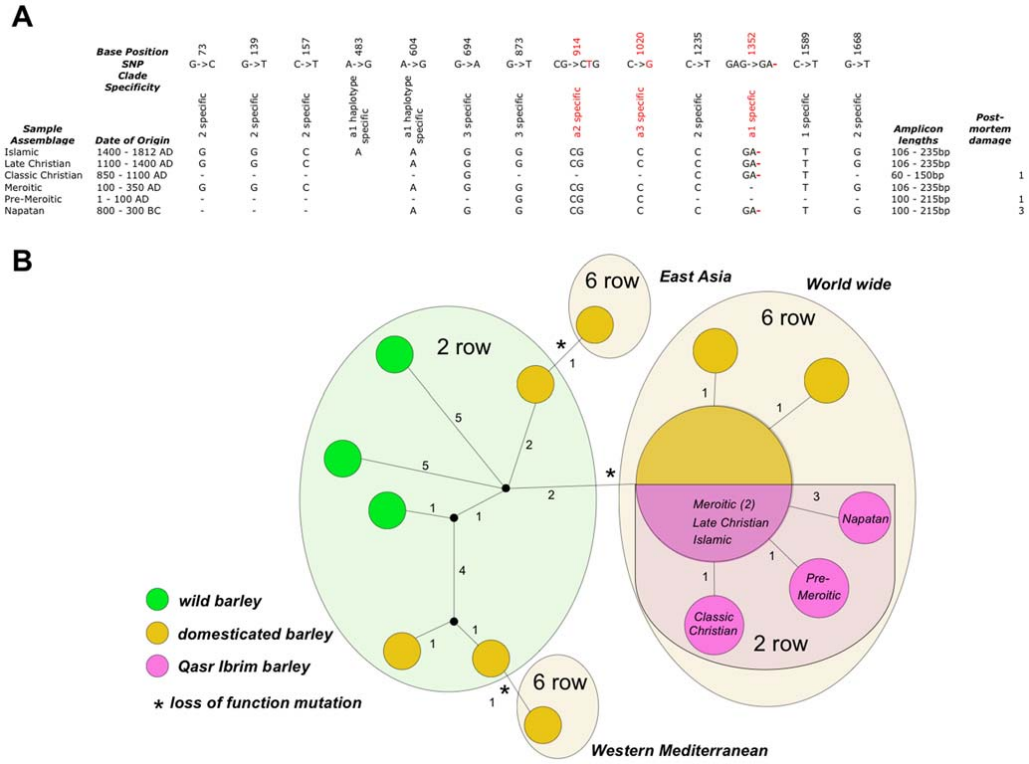
Characterisation of *Vrs1* locus in Qasr Ibrim barley. *(A)* Analysis of Qasr Ibrim barley for haplotype specific polymorphisms [9]. SNPs coloured in red indicate *vrs1* loss of function mutations.–indicates where sequencing data were not available. *(B)* Phylogenetic tree of *Vrs1* alleles indicating the position of the Qasr Ibrim barley within the vrs1.a1 clade.

We amplified the *Vrs1* gene in a series of amplicons that ranged in size from 64–235 base pairs (bp). A block of two amplicon targets which spanned an AT rich region between bases 205–461 did not amplify from any of our samples. All the remaining amplicon targets were amplified from the Islamic and Late Christian samples (200 and 1000 years BP respectively). Amplicons were produced containing thirteen single nucleotide polymorphisms (SNPs) previously observed in [9] from the Meroitic (1450–1800 years BP) sample strata (Fig. 3A). Eleven of these thirteen SNPs were recovered from the Classic Christian (1000–1450 years BP) and Napatan (2400–2900 years BP) sample strata and 5 SNPs (including the 2 *vrs1.a1* clade specific SNPs) were recovered from the Pre-Meroitic (1900–2000 years BP) stratum sample (Figure S3).

To our surprise a single base pair deletion in exon 3 causing a frame shift and the loss of function associated with the *vrs1.a1* worldwide clade for the six-row condition [9] was present in all the samples we tested. Furthermore, the remaining phylogenetically informative sites placed the Qasr Ibrim *vrs1* sequences in clade *vrs1.a1*, (Fig. 3). Eight additional sample specific mutations were found in introns (Table S1, Figure S4). These were all G->A and C->T mutations, consistent with post-mortem damage common of ancient DNA [23]–[26] and are therefore unlikely to represent allelic variants. It is notable in this respect that the oldest sample (Napatan) had the most such mutations and samples from the most recent strata (Islamic) had none, again consistent with base modification over time. The sample from the Classic Christian stratum does not follow the same time-dependent relationship seen in the other samples in terms of the amount of DNA recovered and the number of post-mortem changes. This result could be due to a different diagenetic history in this sample (perhaps the sample was exposed to excessive heat from cooking, for instance), or these barley grains may in fact have originated in an earlier stratum.

### Qasr Ibrim barley was derived from a six-row ancestor

**Figure 4:**
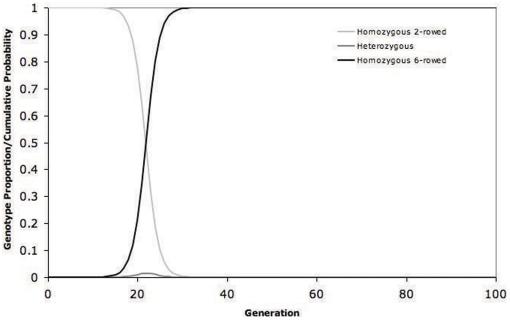
Six-row introduction to two-row populations. Modelling of generations to fixation of recessive six-rowed alleles in homozygous two-rowed barley crops by automatic selection using realistic constraints (Population size: 10^6^; Out-crossing rate: 2%; Six- to two-rowed fecundity ratio: 2.0) the a six-row individual is introduced into a population of 10^6^ two-row plants.

The barley at Qasr Ibrim carries the non-functional *vrs1* allele and therefore is derived from an ancestor that was six-row. This unravels at least part of the mystery; the people of Qasr Ibrim did not necessarily import a two-row type from outside of the region, but that this barley was more likely derived from local six-row barleys. Supporting this scenario is the fact that the phenotype found at Qasr Ibrim and its neighbouring sites differs to that found anywhere else outside the region, because of the characeristic development of lateral bracts. As far as we are aware, this is the first report of a two-row form developing from a six-row ancestor, rather than a two-row condition being reasserted from a six-row one through backcrossing with two-row. However, this sequence of evolutionary events adds to the mystery considerably. Six-row barley produces 1.5–2.0 times as many grains as two-row [13], and has the potential to produce threefold more grains. Consequently, six-row barley has a natural advantage in the population relative to two-row; one would not expect a two-row variety to prevail under normal neutral circumstances. The graph in Fig. 4 demonstrates the output of a model in which a six-row individual is introduced into a population of 10^6^ two-row plants with the properties of barley (2% out-crossing and a six- to two-row fecundity ratio of 2.0). Under these conditions (2% out-crossing and a six- to two-row fecundity ratio of 2.0) the *vrs1* allele conferring the six-row condition would be expected to sweep through the population very rapidly, within the lifetime of a single farmer. The rise of six-row types in early agriculture may be simply explained by fecundity with no selective pressure, conscious or unconscious, derived from farming practice. Under these conditions it is not possible for two-row barley to become established in a six-row population unless the mortality ratio of two to six-row grains is greater than the fecundity ratio of six to two-row; each grain of six-row must be at least twice as likely to die as each two-row grain. A switch from six to two-row would have required extreme selective pressure in favour of the 2-row condition, with a selection coefficient (*s*) equal to a value of 2.

The consistency of the two-row phenotype throughout all the strata spanning three millennia indicates that the mechanism of lateral floret inhibition is more likely to be genetic, not environmental. Consequently, the two-row condition has probably resulted from a gain of function mutation at another locus that reasserted the two-row condition from a six-row ancestor. It is known that the *int-c* gene, which has not yet been isolated and sequenced, can interact with *Vrs1* to modify phenotype [27]–[30], but this is an unlikely candidate to have obtained such a gain of function and be causative of the Qasr Ibrim phenotype since it is thought to interact with the functional *Vrs1* product [29]. Alternatively, a novel mutation at another unknown gene may have been responsible for reasserting lateral floret inhibition.

The type of selection pressure that caused the shift from six-row to two-row is currently a matter for speculation. It is unlikely that it came from conscious action by farmers since such an explanation requires a premeditated awareness of an outcome that, without experience, the farmers could not have had. Alternatively, there may have been a natural selection pressure that strongly favoured the two-row condition. One such possible cause we are currently investigating is water stress. Qasr Ibrim is located in the upper Nile which is very arid relative to the lower Nile where six-row remains are found, and studies have shown that two-row can survive water stress better than six-row [31], [32].

### Local adaptation at Qasr Ibrim

Perhaps the most striking feature about the Qasr Ibrim barley is that successive cultures have the same distinctive two-row phenotype. The discordant combination of phenotype and anomalous *vrs1* genotype is consistent throughout all strata suggesting that the same barley was grown *in situ* despite the cultural transitions, some of which were peaceful, and others more forcible. It may have been the case that six-row varieties of barley were imported with new cultures, but performed poorly against the indigenous two-row phenotype, which itself was ultimately derived from a six-row ancestor. Whatever the reason, successive cultures appear to have adopted the preceding culture's barley agriculture. If this was the case, the Qasr Ibrim barley may represent a ‘lost variety’ that was cultivated continually for thousands of years. Such a long period of cultivation in the same location would give opportunity for the crop to become locally adapted. Primitive landraces of wheat and barley in Europe and maize in South America have shown phylogeographic evidence associated with the Neolithic expansion, and later population movements that are dated to past millennia [33]–[36]. In order for such ancient human movements to be evident, these crops must have existed *in situ* for thousands of years. Under such circumstances adaptation to local environmental conditions would be expected, as appears to be borne out at Qasr Ibrim. A tendency for local adaptation in primitive varieties has important implications in two ways. Firstly, such strong selection pressure is likely to have affected many genes in terms of adaptation, which if identified through archaeogenetics could confirm the nature of the selection pressure and be valuable in the development of new varieties. Secondly, such temporal stability offers an insight into how past cultures are likely to have interacted in terms of adopting indigenous agriculture or importing agriculture with new cultures.

## Materials and Methods

### Archaeobotanical material

Barley samples were collected during excavations at Qasr Ibrim between 1984 and 1986.

### Ancient DNA extraction

DNA extraction was carried out in a dedicated, chambered, ancient DNA extraction laboratory using suitable precautions to avoid contamination by foreign contaminants. This laboratory had not been previously used to extract DNA from modern barley samples and was physically removed from areas where PCR is carried out. Extraction blanks were carried out in parallel with each sample extraction to ensure authenticity. DNA was extracted from 3–6 seeds, ground in a mortar and pestle, and incubated in 1 ml of CTAB buffer (2% cetyltrimethylammoniumbromide [CTAB], 0.1 M trishydroxymethylaminomethane [Tris-HCl] pH 8.0, 20 mM ethylenediaminetetraacetic acid [EDTA], 1.4 M sodium chloride) [37] mixed by agitation for 24 hours at 37°C. The DNA was purified by chloroform:isoamyl alcohol (24∶1) extraction, concentrated using Amicon® concentrators (Centricon® plus-70 with a 30 kDa Ultracel-PL membrane; Millipore) and further purified using a DNeasy® silica column (Qiagen). Double stranded DNA was quantified using a Qubit® Fluorometer (Invitrogen).

### Sequencing of *vrs1* locus

Primer pairs (Text S1) were designed to amplify overlapping segments of *Vrs1*, up to 235 bp in length, capturing known SNPs (Figure S4). DNA amplifications were carried out in 20 µL reactions containing 15 mM Tris-HCl pH 8.3, 50 mM KCl (pH 8.0), 2 mM MgCl_2_, 100 µM of each dNTP, 0.5 U Platinum® Taq DNA polymerase (low DNA; Applied Biosystems), 0.65 µM of each primer and 50–100 pg of the extracted DNA product. PCR and extraction blank reactions were carried out in parallel to the sample PCRs. Thermocycling conditions were as follows: 94°C for 3 min, then 70 cycles of 94°C for 30 s, 60°C for 1 min and 72°C for 1 min, followed by 7 min at 72°C. Blanks and amplification products were confirmed by gel electrophoresis in 2% agarose stained with gel red. QIAquick® (Qiagen) columns were used to purify the PCR products. Sequencing was performed using Big Dye terminator v3.1 Cycle Sequencing kit (Applied Biosystems) and the products separated on a 3130xl Genetic Analyzer at the Genomics Laboratory, HRI, University of Warwick. Sequences have been deposited in GenBank, accession numbers GQ268912–GQ268917.

### *Vrs1* population model

Expectations of fixation of dominant two- and recessive six-rowed alleles in crop populations were calculated by modelling the average number of homozygous and heterozygous individuals at each generation in a population of diploid plants with an initial single heterozygous individual. Harvest seed proportions were predicted with conservative (20%) and realistic (2%) panmictic out-crossing rates [38] and fecundity ratios between six-and two-rowed plants of 1.5 and 2.0 [13]. Seed for each generation were drawn at random from the previous harvest and cumulative probability of extinction through stochastic harvest sampling was calculated at each generation.

## Supporting Information

Figure S1 Barley spikelet morphology from Qasr Ibrim. Abbreviations: fs (fertile spikelet), slb (sterile lateral bract), g (glume), r (rachis). Scale: divisions = 1 mm. a. Unattached spikelet and bract, b. Spikelet and bract attached to rachis. The central fertile spikelet contains a barley grain, sterile lateral bracts do not. The ventral groove of the grain remains untwisted, typical of two-row barley rather than six-row. The resulting barley ear has only two rows of grains
(5.82 MB TIF)

Figure S2 Recoverable archaeological DNA quantity over time. DNA extracted per Barley seed plotted against the years BP of the assemblage from which each sample was recovered. Error bars indicate the range of years BP of the strata from which each sample was taken.
(0.23 MB EPS)

Figure S3 Qasr Ibrim barley amplifications of Vrs1. Regions of the Vrs1 locus amplified (indicated in green) from archaeobotanical remains of barley from Qasr Ibrim and the location of priming sites and the phylogenetically informative SNPs described by (9). Position of exons in Vrs1 shown.
(0.23 MB EPS)

Figure S5 Vrs1 sequence alignment. Alignment of Qasr Ibrim barley sequences against published Vrs1 alleles. The vrs1.a1 clade single base-pair deletion can be seen at position 1353.
(5.02 MB EPS)

Table S1 Qasr Ibrim Barley DNA studies summary table. DNA extracted per seed, length range of amplicons and C->T and G->A base pair modifications recovered from each of the Qasr Ibrim assemblages.
(0.27 MB EPS)

Text S1 Priming sites. Priming sites used to amplify 1747 bp of the vrs1 locus from the archaeobotanical remains of barley from Qasr Ibrim. * Indicates previously published (9).
(0.03 MB DOC)
