# Supplementary figures and images for: Archaeogenetic Evidence of Ancient Nubian Barley Evolution from Six to Two-Row Indicates Local Adaptation
Source: PLoS One. 2009 Jul 22;4(7):e6301. doi: 10.1371/journal.pone.0006301 (PMC2707625; doi:10.1371/journal.pone.0006301)

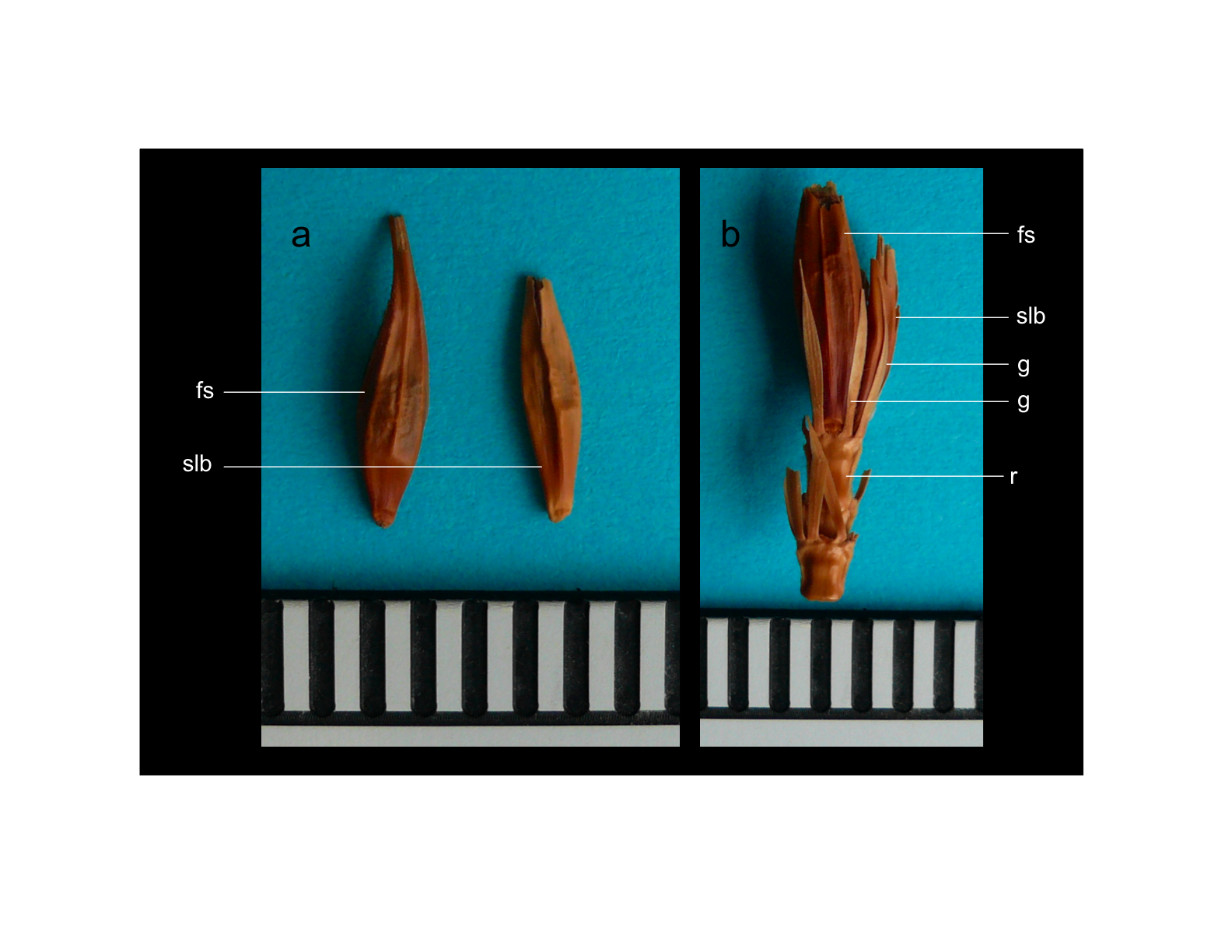

Supplement: Figure S1 — Barley spikelet morphology from Qasr Ibrim. Abbreviations: fs (fertile spikelet), slb (sterile lateral bract), g (glume), r (rachis). Scale: divisions = 1 mm. a. Unattached spikelet and bract, b. Spikelet and bract attached to rachis. The central fertile spikelet contains a barley grain, sterile lateral bracts do not. The ventral groove of the grain remains untwisted, typical of two-row barley rather than six-row. The resulting barley ear has only two rows of grains (5.82 MB TIF) [file pone.0006301.s001.tif]
