# Supplementary material for: Archaeogenetic Evidence of Ancient Nubian Barley Evolution from Six to Two-Row Indicates Local Adaptation
Source: PLoS One. 2009 Jul 22;4(7):e6301. doi: 10.1371/journal.pone.0006301 (PMC2707625; doi:10.1371/journal.pone.0006301)
Supplement: Text S1 — Priming sites. Priming sites used to amplify 1747 bp of the vrs1 locus from the archaeobotanical remains of barley from Qasr Ibrim. * Indicates previously published (9). (0.03 MB DOC) [file pone.0006301.s006.doc]

5' GTCATAACTCGGCAAACATAG 3' *

5' TATCTAGAGGAACTCGATGAACTT 3'

5' GTTCTTCCTTTCACCGAGTAC 3'

3' GTACTCGGTGAAAGGAAGAAC 5'

5' GCGTGTACTAGGCAAATATATG 3'

3' ATGTATTTGTCAAGTTTGGTCCA 5'

5' CTCCGAATGAAATGAACTCTGC 3'

3' GCAGAGTTCATTTCATTCGGAG 5'

5' CTCAGGTCAATGCTAATGTGG 3'

3' CCACATTAGCATTGACCTGAG 5' *

5' TGGATCGGAAAGCACTCAGC 3'

3' GCTGAGTGCTTTCCGATCCA 5'

5' CTTGCCGTTGGGTACCTCT 3'

3' CCAGGGAGAGACCTAGGG 5'

5' CCTCCTTCAACTAGTGCTTTG 3'

3' CAAAGCACTAGTTGAAGGAGG 5'

5' GAGCACACCAACAGGCAACA 3'

3' GGAGGGGAGACACGGTAG 5'

5' TCAGATCCGAACCGAAAGCAT 3'

3' ATGCTTTCGGTTCGGATCTGA 5'

3' GCGAAGAAAGTCGTGTCCAC 5'

5' CAGCAAGCAGAGGGCGC 3'

3' GCGCCCTCTGCTTGCTG 5'

5' GAGGGGATGGTGACGGTG 3'

3' CACCGTCACCATCCCCTC 5'

5' GCCGAGATTCTGGAGCTGA 3'

5' GAGAACGAGGTATGCTTGCTC 3'

3' CAGCGCCATATGTAAGCCAG 5'

5' GAGAGACTGGGAGCGACTG 3'

3' CCCAGCTGCCGACCTGAG 5'

5' CATGAATTAGAGTTTATGCTGG 3'

3' AACACTCGACCACGCTGCTA 5'

5' ATAGCCGAGATAGCTGCTGC 3'
